# Nutritional status and quality of life of cancer patients needing exclusive chemotherapy: a longitudinal study

**DOI:** 10.1186/s12955-017-0660-6

**Published:** 2017-04-27

**Authors:** Sebastien Salas, Sophie Mercier, Benjamin Moheng, Sandrine Olivet, Marie-Eve Garcia, Sophie Hamon, Camille Sibertin-Blanc, Florence Duffaud, Pascal Auquier, Karine Baumstarck

**Affiliations:** 1 Assistance Publique Hôpitaux de Marseille, Timone Hospital, Department of adult oncology, Marseille, 13005 France; 2 CRO2, Aix Marseille Université, Marseille, 13284 France; 3 EA 3279 Self-Perceived Health Assessment Research Unit, Aix Marseille Université, APHM, 27 bd Jean Moulin, Marseille cedex 05, F-13385 France; 4 Assistance Publique Hôpitaux de Marseille, Multidisciplinary Oncology and Therapeutic Innovations Department, Aix Marseille University, Centre d’Investigation Clinique, Marseille, 13385 France; 5 Assistance Publique Hôpitaux de Marseille, Division of Gastroenterology, Aix-Marseille University, Marseille, 13385 France; 6 Assistance Publique Hôpitaux de Marseille, Department of Digestive Oncology, Aix-Marseille University, Marseille, 13385 France

**Keywords:** Nutrition, Cancer, Chemotherapy, Quality-of-life

## Abstract

**Background:**

The aims of this study were to report nutritional status in a large panel of patients with cancer requiring exclusive chemotherapy and to study the influence of nutritional status on their quality of life (QoL).

**Methods:**

This work was a longitudinal cohort study performed at a French university teaching hospital. Eligible patients were individuals with a cancer needing treatment based on exclusive chemotherapy. Three work-ups were performed: i) before the administration of the first course of chemotherapy: T1, ii) before the administration of the second (for patients with 3 planned courses) or third (patients with 6 planned courses) course: T2, and iii) before the administration of the last planned course: T3. The following data were collected: general health (performance status) and nutritional status (weight, anorexia grading, albuminemia, pre-albuminemia, and C-reactive protein) and QoL.

**Results:**

The nutritional status of patients with cancer was preserved. Functional impairment, the presence of anorexia, the palliative nature of the chemotherapy, and an elevated C-reactive protein dosage were independent predictive factors of a lower QoL among patients assessed at the end of chemotherapy.

**Conclusions:**

Although larger studies should corroborate these findings, clinicians may include this information in the management of patients with cancer requiring exclusive chemotherapy to identify the most vulnerable patients.

**Trial registration:**

Current controlled trials NCT01687335 (registration date: October 6, 2011).

**Electronic supplementary material:**

The online version of this article (doi:10.1186/s12955-017-0660-6) contains supplementary material, which is available to authorized users.

## Background

Advances in oncology have promoted patient survival and have consequently revealed previously insignificant complications, such as malnutrition. Specifically, malnutrition is a very common problem among oncology patients [1, 2], and cancer malnutrition, defined as an unbalanced nutritional intake/needs ratio leading to functional consequences, is multifactorial and related both to the disease and the anti-tumor treatments: i) reduced intake [3] due to multiple functional disorders (anorexia, nausea and vomiting, malabsorption, anxiety, pain, etc.) and ii) increased needs due to hypermetabolism. Moreover, inadequate nutrition is associated with a poorer prognosis/survival [1, 4–6] and deteriorated quality of life (QoL) [7, 8].

Chemotherapy is one of the most aggressive cancer treatments and may have serious adverse effects. Because malnutrition is considered increasingly important in the setting of cancer, several (French and international) groups have published recommendations and guidelines for including nutritional management in the global management of this disease [9–11], specifically for patients requiring chemotherapy [12]. For example, the European Society for Clinical Nutrition and Metabolism (ESPEN) advocates systematic nutritional assessment before and during all exclusive chemotherapy procedures to identify nutritional deficits early and to plan targeted actions [12].

To our knowledge, few robust and reliable studies concerning the nutritional status of patients with cancer who require chemotherapy are available, and the degree to which the international recommendations are applied has not yet been reported. In this study, we first report the nutritional status of a large panel of patients with cancer requiring exclusive chemotherapy and report the influence of nutritional status on the patients’ QoL.

## Materials

### Design and setting

This longitudinal prospective cohort study was performed in a French university teaching hospital (Timone Hospital, Marseille) and was funded by institutional grants from the French 2013 Clinical Research Program from Assistance Publique-Hôpitaux de Marseille (AP-HM, France). Patients were recruited from the four adult oncological departments. Methodological support was provided by the Clinical Research Unit (Unité Aide Méthodologique à la Recherche Clinique, AP-HM, France).

### Patients

Patients who had cancer requiring treatment based on exclusive chemotherapy were eligible to be included if they met the following inclusion criteria: age over 18 years, histological diagnosis of cancer, and requiring chemotherapy with curative or palliative intent (planned with 3 separate cures 21 days apart or 6 separate cures 14 days apart). Patients were excluded if they met the following criteria: surgery in the last 4 weeks or radiotherapy in the last 2 weeks.

### Ethics

In accordance with French law, the study protocol was approved by a French Ethics Committee (Comité de Protection des Personnes Sud Mediterranée 1 Ref: 1126/09 14 2011; IDRCB: 2011/A00631/40). All patients provided written informed consent (Trial registration: current controlled trials NCT01687335 (registration date: October 6, 2011)).

### General schedule

After the baseline assessment, three supplementary work-ups were performed for each participant: i) before the administration of the first course of chemotherapy: T1, ii) before the administration of the second (for patients with 3 planned courses) or third (patients with 6 planned courses) course of chemotherapy: T2, and iii) before the administration of the last planned course: T3.

### Data collection

- At baseline: socociodemographics (age, gender, and marital status); tumor: localization, staging; health status (performance status according to WHO classification, usual weight and body mass index (BMI)); notion of palliative care stage according to WHO definition (WHO pain ladder http://www.who.int/cancer/palliative/painladder/en/.)
- At each time point: general health status (performance status); nutritional status assessed using: 1. Clinical parameters: weight and BMI, subjective grading of anorexia (on a scale from 0 to 4 indicating no anorexia to severe anorexia). 2. Biological parameters: albuminemia, pre-albuminemia, C-reactive protein (CRP), and plasma orosomucoid concentration.
- The degree to which the nutritional recommendations of the European Society for Parenteral and Enteral Nutrition were applied was assessed using four criteria: 1) one dietetic visit during the chemotherapy course (yes/no), 2) nutritive supplementation for patients with malnutrition and/or patients with caloric requirements not covered (yes/no), 3) oral caloric supplementation (when oral nutrition was possible) for patients with malnutrition and/or for patients with caloric requirements not covered (yes/no), and 4) artificial enteral or parenteral nutritive supplementation (when oral nutrition was not possible) for patients with malnutrition and/or for patients with caloric requirements not covered (yes/no). Parenteral nutrition was offered in cases of contraindication to enteral feeding. Two degrees of adequacy were defined: strict adequacy (the four criteria were respected) and lesser adequacy conformed to the last three factors, irrespective of the adequacy with the first factor (dietetic advice)). Adequacy was assessed at each time point (T1, T2, and T3 adequacy) and at the end of chemotherapy (global adequacy).
- QoL was assessed only at T1 and T3 using the European Organization for Research and Treatment of Cancer (EORTC) QLQ-C30 questionnaire, which is a 30-item questionnaire consisting of five functional scales (physical, role, emotional, cognitive, and social), nine symptom scales and single-symptom items, and a global health status scale [13]. The scores on each scale/item range from 0 to 100. A high score on the functional scale represents a high/healthy level of functioning, a high score on the global health status scale represents a high QoL (the scores of the symptom scales were not analyzed).

### Definitions of interest

Malnutrition was defined according to the following parameters [1]:

- clinical definition based on body mass index (BMI) (malnutrition when BMI < 21 kg/m^2^)
- biological definitions based on albuminemia (<30 g/l) (late marker of undernutrition) and pre-albuminemia (early marker of undernutrition) (<110 mg/l).

Hypercatabolism was defined as a CRP (>15 mg/l) [1].

The risk of complications related to undernutrition was defined using the Prognostic Inflammatory and Nutritional Index (PINI = (Orosomucoıd x CRP)/(albuminemia x pre-albuminemia).

### Statistical analysis

Descriptive data were obtained from the total sample. Continuous variables are expressed as the means, standard deviations, medians, and interquartile ranges (IQR). Qualitative variables are expressed as percentages. The five functional QoL scores in the sample were compared between T1 and T3 using paired Student’s *t*-test. QoL scores at T3 were tested with the following parameters using Student’s *t*-test for qualitative variables and Pearson’s correlation coefficients for continuous variables: i) sociodemographic parameters, ii) nutritional variables at T1, and iii) nutritional variables at T3. Tests for multiple comparisons were performed (Bonferroni). Multivariate analyses using multiple linear regressions were performed to identify variables potentially predictive of QoL at T3 (5 function scores). Each function dimension was considered as a separate dependent variable. Variables relevant to the models were selected by applying a threshold *p*-value <0.1 in the univariate analysis. The final models expressed the standardized beta coefficient. The coefficient represents the change in the standard deviation of the dependent variable (QoL) resulting from a change in one standard deviation of the independent variables. The independent variables with the highest standardized beta coefficient were the variables with the greatest relative effect on quality of life. All tests were two-sided. Significance was defined as p <0.05. All statistical analyses were performed using the SPSS software package, version 20.

## Results

### Sample characteristics at baseline

Between May 2011 and August 2014, 102 patients were included. The sex ratio was nearly 1, and the median age was 61 (interquartile range 49–69). The baseline characteristics of the sample are shown in Table 1. Head and neck carcinomas, sarcomas and gynecologic cancers were the three most frequent localizations. Based on the BMI, 10% of the patients were undernourished at inclusion. A T2 and T3, 96 and 91 individuals were assessed, respectively.

**Table 1:** Baseline characteristics of the patients (*N* = 102)

		*N* (%) or M ± SD	MD
1. Sociodemographics			
Gender	Women Men	52 (51,0) 50 (49,0)	0
Age		58,4 ± 14,2	1
Marital status	Couple Single	59 (70,2) 25 (29.8)	18
2. Clinical data			
Cancer localization	Head and neck STS Gynecologic Others^a^	22 (21,6) 25 (24,5) 24 (23,5) 31 (30,4)	0
Performans status	0 ≥1	49 (57,6) 36 (42,4)	17
Chemotherapy	Curative Palliative	49 (59,8) 33 (40,2)	20
Usual BMI (kg/m^2^)		22,8 ± 3,1	4
	Malnutrition (BMI<21 kg/m^2^) BMI > = 21 kg/m^2^	10 (10,2) 88 (89,8)	4

*M* ± *SD* mean ± standard deviation, *MD* missing data, *STS* Soft Tissue Sarcoma, *BMI* Body Mass Index: ^a^others: urologic, adenopathy, thyroid, digestive, lung

### Nutritional statuses

The patients’ nutritional characteristics at T1, T2 and T3 are shown in Table 2. The proportion of patients with malnutrition remained constant over time, irrespective of the biological definition, but the proportion of patients with malnutrition according to BMI was higher at T2 (not significant). The global adequacy of the application of nutritional recommendations was reported in 19% and 91% of individuals for the strict definition and less stringent definition, respectively. Regarding the strict definition, the adequacy was 35, 35 and 27% at T1, T2 and T3, respectively. For the less stringent definition, it was 95, 99 and 94% at T1, T2 and T3, respectively.

**Table 2:** Nutritional parameters at the three assessments

		T1	T2	T3
		*N* = 102	*N* = 96	*N* = 91
1. Clinical parameters		*N* (%) or M ± SD	*N* (%) or M ± SD	*N* (%) or M ± SD
Weight (kg)		68,7 ± 13,9	68,5 ± 13,9	68,5 ± 14
BMI (kg/m^2^)^a^		24,2 ± 4,4	24,2 ± 4,4	24,1 ± 4,5
	Malnutrition No malnutrition	15 (16,3) 77 (83,7)	19 (21,1) 71 (78,9)	13 (16,9) 64 (83,1)
Anorexia grading^d^	0 > = 1	89 (90,8) 9 (9,2)	82 (89,1) 10 (10,9)	75 (90,4) 8 (9,6)
Exclusive oral feeding		91 (95,8)	85 (95,5)	80 (93)
Nutritive supplements		3 (3,2)	3 (3,4)	7 (8,1)
2. Biological parameters				
Albuminemia (g/l)^b^		34,4 ± 4,4	34,7 ± 4,8	34,9 ± 5,5
	Malnutrition No malnutrition	12 (13,2) 79 (86,8)	12 (16,7) 60 (83,3)	12 (15,6) 65 (84,4)
Prealbuminemia (mg/l)^c^		253,3 ± 72,0	255,3 ± 82,7	273 ± 170,1
	Malnutrition No malnutrition	2 (2,4) 80 (97,6)	4 (6,3) 60 (93,7)	2 (3,3) 58 (96,7)
CRP (mg/l)	Median (IQR^e^)	6,2 (2,0-17,1)	2,5 (1,0-16,8)	3 (1,0-12,3)
PINI score	Median (IQR^e^)	0,5 (0,1-1,8)	0,2 (0,1-3,1)	0,2 (0,1-0,7)

T1 first course of chemotherapy; T2 the second course of chemotherapy; T3 the last planned course of chemotherapy

*BMI* Body Mass Index, *CRP* C-reactive protein, *PINI* Prognostic Inflammatory and Nutritional Index

^a^malnutrition if BMI < 21 kg/m^2^; ^b^malnutrition if albuminemia < 30 g/l; ^c^malnutrition if prealbuminemia < 110 mg/l; ^d^0: no anorexia, > = 1: 1 to 3 anorexia grading; ^e^interquartile range

### Quality of life

**Fig. 1:**
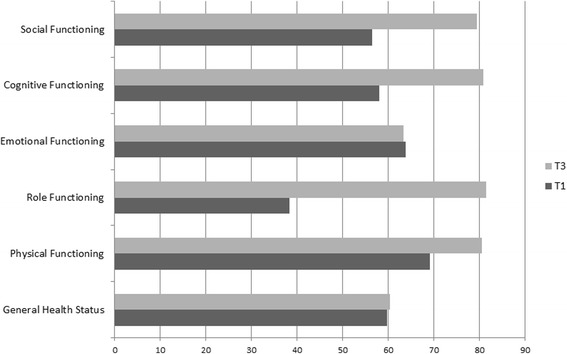
Quality of life change between the beginning and the end of the planned chemotherapy. T1 before the administration of the first course of chemotherapy. T3 before the administration of the last planned course of chemotherapy

The functional scores of EORTC QLQ C30 are shown in Fig. 1. Cognitive function scores were lower at T3 than at T1.

### Potential predictive role of nutritional status on quality of life

The results of the univariate analysis are available in Additional file 1: Table S1. Multivariate models were built to identify independent variables linked to QoL scores at T3. After adjustment based on five factors, three factors were independently linked to global health status at T3: performance status, anorexia grade and CRP. Moreover, receiving palliative chemotherapy was an independent predictive factor of three scores: physical, role and emotional functions. BMI was an independent factor of cognitive function, and anorexia was an independent factor of social function. All results are detailed in Table 3.

**Table 3:** Factors associated to quality of life scores at T3

			β	p-value
Global health	Performans status	Ref (0)	−0,348	**0,011**
	Anorexia grading	Ref (0)	−0,384	**0,005**
	Prealbuminemia	Ref (<0.110)	0,071	0,679
	CRP		−0,496	**0,011**
	PINI		0,431	0,082
Physical function	Chemotherapy	Ref (palliative)	0,384	**0,002**
	Performans status	Ref (0)	−0,236	0,060
	BMI classes	Ref (<21)	−0,118	0,309
	Anorexia grading	Ref (0)	0,005	0,968
Role function	Chemotherapy	Ref (palliative)	0,370	**0,011**
	Performans status	Ref (0)	−0,111	0,459
	Prealbuminemia	Ref (<0.110)	0,145	0,350
	Anorexia grading	Ref (0)	−0,098	0,517
Emotional function	Chemotherapy	Ref (palliative)	0,355	**0,002**
	BMI classes	Ref (<21)	−0,158	0,168
Cognitive function	Chemotherapy	Ref (palliative)	0,093	0,446
	BMI classes	Ref (<21)	−0,304	**0,023**
	Anorexia grading	Ref (0)	0,025	0,847
Social function	Performans status	Ref (0)	−0,058	0,631
	Weight change		0,147	0,201
	BMI classes	Ref (<21)	−0,020	0,864
	Anorexia grading	Ref (0)	−0,369	**0,003**

*BMI* Body Mass Index, *CRP* C-reactive protein, *PINI* Prognostic Inflammatory and Nutritional Index, *β* beta standardized coefficient, *Ref* reference modality, Bold values: *p*-value <0.05

## Discussion

This study investigated the nutritional status of a large sample of patients with cancer requiring exclusive chemotherapy. Although international institutions promote the assessment and management of malnutrition [12], few studies have specifically examined malnutrition in these patients. Here, the large majority of patients exhibited a satisfactory nutritional status. Previous studies reported higher proportions of patients with cancer who were undernourished, but these studies examined more heterogeneous samples [1, 2]. Malnutrition was diagnosed in only 2 to 16% of our patients according to clinical (BMI) and biological parameters (albuminemia and pre-albuminemia). This divergence may be due to the selection of individuals. Specifically, only patients of good general status are eligible to receive chemotherapy, and such patients are consequently less malnourished, whereas patients who receive surgery or radiotherapy are more malnourished. Unsurprisingly, these indicators slightly worsened during the course of chemotherapy, particularly around the second course. Caution is required during this period because the side-effects of the first course may worsen the patient’s nutritional status. The low proportion of undernourished patients may also be due to the fact that recommendations regarding nutritional management were largely followed, at least the recommendations according to a definition less stringent than that of the ESPEN (lacking one criterion: one dietetic visit during the chemotherapy course).

We herein reported, for the first time, that consensual nutritional recommendations in this specific population are satisfactory. Specifically, the proportion of patients for whom nutritional management was appropriate exceeded 90% at each assessment based on the less stringent definition of adequacy (see methods section). However, 65 to 80% of the patients were not managed in conformity with the stricter recommendations, mainly due to the absence of a dietetic consultation, which should be prescribed for patients identified by clinicians as being the most vulnerable.

Although gender and age are often linked to QoL, we found no such association in our sample. As expected, the type of chemotherapy impacted some QoL domains: due to their advanced disease status, patients receiving palliative chemotherapy had a lower QoL than patients receiving curative chemotherapy. Furthermore, performance status, which reflects functional impairment, seems to be a sensitive indicator of the physical QoL dimension, as previously reported [8, 14]. Among the clinical parameters of nutrition, anorexia was identified to impact the QoL the most. Although anorexia or malnutrition may restrict individuals in their various social activities, other facets of QoL may also exacerbate fatigue and mood disorders. The predictive role of the biological parameters of nutrition is more ectopic. Specifically, albuminemia and QoL did not correlate, but pre-albuminemia, CRP and the PINI scores were related to lower QoL scores. Although the predictive role of biological nutritional factors in the response to radio-chemotherapy has already been demonstrated [4], few data are available on the predictive value of these biological nutritional factors on the QoL of patients treated exclusively with chemotherapy. To our knowledge, only one study focusing on the specific role of CRP, which is known to be a marker of systemic inflammation and hypercatabolism, was shown to predict QoL [15]. A multivariate approach confirmed that the following parameters independently predicted QoL: performance status, anorexia, type of chemotherapy and CRP level. Although larger studies should corroborate these findings, clinicians may already include this information in the management of patients with cancer requiring exclusive chemotherapy to identify the most vulnerable patients. For example, forthcoming anorexia drugs, such as anamorelin, an oral ghrelin-receptor agonist with appetite-enhancing and anabolic activity [16], should improve QoL of these individuals. Specifically, anamorelin had a favorable clinical response profile in patients with cancer anorexia-cachexia syndrome [17].

In addition to the more traditional markers of undernutrition, clinicians should be more attentive to changes in CRP, a well-known marker of hypercatabolism, in this specific population. Lastly, the PINI score may serve as a new predictive factor of QoL, although our data did not confirm its role in the multivariate analysis.

### Limitations

This work was also subject to limitations. First, we adopted the BMI, but not weight loss, as a criterion for malnutrition. In Western countries, where being overweight or obese is prevalent among patients with cancer, using weight loss may be more appropriate to diagnose malnutrition. Recently, ESPEN proposed new diagnostic criterion that may be used in this group of patients to assess discrepancies in evaluating malnutrition.

Another limitation of this study is the representativeness of the sample, which did not differ in terms of age and sex ratio compared to a comparable French population of patients that is available in the 2014 report of the French National Institute of Cancer (INCA Institut national contre la cancer) [18]. However, the proportion of head and neck carcinomas and sarcomas was high, whereas the three most common cancers treated in France are digestive, genital, and hematological malignancies [18]. This discrepancy is due to the specific populations that are managed at the participating centers.

Moreover, the small sample size precludes a deeper investigation of associations with the QoL, especially investigations regarding the nature of chemotherapy, the combination of chemotherapy and the notion of radiotherapy and/or surgery before inclusion.

Lastly, future studies comparing clinical guidelines in other countries (United States and other countries in Europe) to the French guidelines would provide additional and useful information.

## Conclusions

The nutritional status of patients with cancer requiring exclusive chemotherapy was relatively preserved. Functional impairment, the presence of anorexia, the palliative nature of the chemotherapy and an elevated CRP dosage appear to be independent predictive factors of QoL in patients at the end of chemotherapy.

## Additional files

Additional file 1: Table S1. Relationships between QoL scores of functioning scales (EORTC QLQ C30) at T3 and sociodemographic and nutritional status. (DOCX 54 kb)

Additional file 2: Dataset. (XLS 470 kb)

## Abbreviations

BMI: Body mass index
CRP: C-reactive protein
EORTC: European organization for research and treatment of cancer
ESPEN: European society for clinical nutrition and Metabolism
IQR: Interquartile ranges
PINI: Prognostic Inflammatory and Nutritional Index
QoL: Quality of life
