# Supplementary material for: Nutritional status and quality of life of cancer patients needing exclusive chemotherapy: a longitudinal study
Source: Health Qual Life Outcomes. 2017 Apr 27;15:85. doi: 10.1186/s12955-017-0660-6 (PMC5408409; doi:10.1186/s12955-017-0660-6)
Supplement: Supplementary file 1 — Relationships between QoL scores of functioning scales (EORTC QLQ C30) at T3 and sociodemographic and nutritional status. (DOCX 54 kb) [file 12955_2017_660_MOESM1_ESM.docx]

**Supplementary table 1. Relationships between QoL scores of functioning scales (EORTC QLQ C30) at T3 and sociodemographic and nutritional status**

|  |  | Global | Physical | Role | Emotional | Cognitive | Social |
| --- | --- | --- | --- | --- | --- | --- | --- |
|  |  | health | Function | Function | Function | Function | Function |
|  |  | M±SD/R | M±SD/R | M±SD/R | M±SD/R | M±SD/R | M±SD/R |
| **Baseline** |  |  |  |  |  |  |  |
| Gender | Women | 66,7±19,3 | 75,6±21 | 70,5±26,4 | 71,5±21,6 | 73,9±27 | 75±27,3 |
|  | Men | 59,4±27,7 | 73,8±24,7 | 65±34,2 | 78,8±18,7 | 83,3±24,5 | 76,5 ±29,3 |
|  | p | 0,18 | 0,73 | 0,41 | 0,10 | 0,10 | 0,81 |
| Age | R | -0,10 | -0,06 | -0,04 | 0,01 | -0,04 | -0,12 |
|  | p | 0,37 | 0,61 | 0,7576 | 0,94 | 0,69 | 0,29 |
| Marital status | Couple | 63,8±25 | 75,5±21,7 | 66,3±30,3 | 73,1±21,2 | 77,9±27,2 | 77±26,9 |
|  | Single | 61,5±23,4 | 71,1±25,8 | 69,4±31 | 78,5±20,1 | 75±26 | 72,2±32,1 |
|  | p | 0,70 | 0,44 | 0,68 | 0,29 | 0,66 | 0,51 |
| Chemotherapy | Curative | 66,7±26,9 | 82±20,6 | 73,8±27,7 | 79,8±21,1 | 82,3±24,2 | 77,3±29 |
|  | Palliative | 59,2±18,9 | 64,5±20,6 | 58,3±32,9 | 66,1±18 | 72±26,5 | 76,2±25 |
|  | p | 0,20 | **0,001^(^*^)^** | **0,033** | **0,005^(^*^)^** | 0,091 | 0,70 |
| **T1** |  |  |  |  |  |  |  |
| Performans status | 0 | 67,4±22,2 | 79,3±22 | 68,1±32,4 | 75,9±21,2 | 79,3±25,1 | 76,4±29,1 |
|  | ≥ 1 | 57,3±26,4 | 67,3±23,1 | 67,2±27,7 | 73,9±20,7 | 74,2±28,8 | 74,7±27,8 |
|  | p | 0,073 | **0,024** | 0,898 | 0,686 | 0,408 | 0,797 |
| BMI | R | -0,14 | -0,23 | -0,21 | -0,20 | -0,09 | -0,31 |
|  | p | 0,22 | **0,038** | 0,60 | 0,07 | 0,44 | **0,005^(^*^)^** |
| Albuminemia | Malnutrition | 58,3±28,9 | 73,3±21,8 | 70±40,7 | 81,7±16,6 | 81,7±22,8 | 78,3±31,5 |
|  | Not malnutrition | 64,1±22,5 | 74,8±22,7 | 68,2±28 | 74,5±20,8 | 77,6±27,1 | 76,8±25,7 |
|  | p | 0,47 | 0,85 | 0,89 | 0,30 | 0,66 | 0,87 |
| Prealbuminemia | Malnutrition | 41,7±35,4 | 70±4,7 | 58,3±58,9 | 58,3±11,8 | 66,7±47,1 | 100±0 |
|  | Not malnutrition | 64,4±23,8 | 75,2±23,9 | 67,7±30,6 | 74,9±21,2 | 76,4±27,3 | 75,6±27,8 |
|  | p | 0,19 | 0,33 | 0,68 | 0,278 | 0,63 | **0,001^(^*^)^** |
| CRP | R | -0,14 | -0,16 | -0,07 | -0,01 | -0,06 | 0,07 |
|  | p | 0,24 | 0,19 | 0,56 | 0,91 | 0,60 | 0,55 |
| PINI score | R | -0,24 | -0,10 | -0,18 | -0,15 | -0,18 | 0,13 |
|  | p | 0,06 | 0,46 | 0,17 | 0,26 | 0,18 | 0,32 |
| Anorexia grading° | 0 | 64,8±23,5 | 76,2±21,8 | 68,4±30,7 | 75,2±20,5 | 80,3±23,7 | 77,5±26,7 |
|  | ≥ 1 | 43,1±17 | 56,7±28,5 | 61,1±25,1 | 72,2±22,8 | 52,8±42,7 | 52,8±37,1 |
|  | p | **0,029** | **0,042** | 0,57 | 0,74 | 0,18 | **0,037** |
| **T2** |  |  |  |  |  |  |  |
| Performans status | 0 | 70,3±24,7 | 83,3±20,3 | 73±33,8 | 76,2±20,9 | 84,3±23,9 | 82,8±23,7 |
|  | ≥ 1 | 60,9±22,5 | 68,9±24,2 | 67,9±27,7 | 76,7±21 | 73,5±28,5 | 70,9±31 |
|  | p | 0,09 | **0,008^(^*^)^** | 0,48 | 0,92 | 0,09 | 0,07 |
| Weight change | R | -0,09 | 0,12 | 0,06 | 0,10 | 0,05 | 0,22 |
|  | p | 0,41 | 0,29 | 0,59 | 0,38 | 0,63 | **0,047** |
| BMI | R | -0,14 | -0,21 | -0,18 | -0,19 | -0,07 | -0,27 |
|  | p | 0,21 | 0,06 | 0,11 | 0,08 | 0,49 | **0,016** |
| Albuminemia | Malnutrition | 60±26 | 73,3±19,9 | 73,3±30,6 | 84,2±16,9 | 83,3±23,6 | 83,3±22,2 |
|  | Not malnutrition | 64,5±24,2 | 74,5±24,7 | 67±29,1 | 74,1±21,9 | 76,3±28,3 | 75,4±28,2 |
|  | p | 0,60 | 0,89 | 0,53 | 0,17 | 0,46 | 0,40 |
| Prealbuminemia | Malnutrition | 38,9±19,2 | 53,3±13,3 | 33,3±16,7 | 69,4±21 | 66,7±33,3 | 72,2±34,7 |
|  | Not malnutrition | 64,6±22 | 74,5±24,3 | 69,6±29,4 | 75,1±22,2 | 76,6±28,3 | 77,2±27,9 |
|  | p | 0,05 | 0,14 | **0,039** | 0,67 | 0,56 | 0,77 |
| CRP | R | -0,31 | -0,20 | -0,17 | -0,01 | -0,0364 | -0,02 |
|  | p | **0,013^(^*^)^** | 0,11 | 0, | 0,92 | 0,78 | 0,86 |
| PINI score | R | -0,31 | -0,08 | -0,20 | -0,10 | -0,15 | 0,08 |
|  | p | **0,018** | 0,57 | 0,13 | 0,45 | 0,27 | 0,54 |
| Anorexia grading° | 0 | 66,3±23,1 | 76,1±22,3 | 70,1±30,2 | 75,6±20,5 | 80,6±24,5 | 79,9±24,9 |
|  | ≥ 1 | 38,9±15 | 62,2±24,7 | 51,9±26,9 | 71,3±21,7 | 59,3±33,5 | 46,3±35,1 |
|  | p | **0,001^(^*^)^** | 0,09 | 0,09 | 0,56 | **0,021** | **<0.001^(^*^)^** |

Bold values p<0.05

(*) p<0.05 after correction for multiple testing

° 0: no anorexia, >=1: 1 to 3 anorexia grading

BMI Body Mass Index; CRP C-reactive protein; PINI Prognostic Inflammatory and Nutritional Index
